# Isolation, Risk Factors, and Antimicrobial Resistance of *Escherichia coli* and *Salmonella* in Diarrheic Calves in Sebeta, Ethiopia

**DOI:** 10.1155/vmi/7166641

**Published:** 2026-09-16

**Authors:** Atinafu Regasa Hailu, Fikru Gizaw Gurmessa, Teshita Edaso Bariso, Shubisa Abera Leliso

**Affiliations:** ^1^ School of Veterinary Medicine, Arsi University, Asella 193, Ethiopia, arsiun.edu.et; ^2^ College of Veterinary Medicine, Samara University, Samara 132, Ethiopia, su.edu.et; ^3^ National Animal Health Institute, Sebeta 04, Ethiopia

**Keywords:** antimicrobial susceptibility, calf diarrhea, *Escherichia coli*, Ethiopia, *Salmonella*

## Abstract

Calf diarrhea is a major constraint to dairy production in Ethiopia, resulting in substantial economic losses and potentially complicated by antimicrobial‐resistant bacterial pathogens. This study aimed to determine the isolation of *Escherichia coli* and *Salmonella* in diarrheic calves, identify associated risk factors, and assess antimicrobial susceptibility patterns. A cross‐sectional study was conducted in calves less than 2 months of age in and around Sebeta Town. The study area and districts were selected purposively, dairy farms were selected by simple random sampling, and diarrheic calves were selected purposively. A total of 129 fecal samples were collected and examined using standard bacteriological techniques and Biolog identification. Antimicrobial susceptibility testing was performed using the disk diffusion method. *E. coli* and *Salmonella* were isolated from 65 (50.3%; 95% CI: 41.6–59.1) and 8 (6.2%; 95% CI: 1.98–10.4) of the 129 samples, respectively. In multivariable logistic regression, sex, age, colostrum feeding, bedding, and housing were significantly associated with increased odds of *E. coli* isolation. For *Salmonella* sex, age, and bedding were significantly associated with increased odds of isolation. Most *E. coli* isolates were susceptible to sulphamethoxazole–trimethoprim (93.85%), polymyxin B (78.46%), gentamicin (76.92%), and ampicillin (73.85%), whereas tetracycline showed the highest resistance (58.46%). Among the 20 *E. coli* isolates exhibiting resistance to two or more antimicrobial agents, three of twenty (15.0%) fulfilled the predefined multidrug resistance criterion. All *Salmonella* isolates were susceptible to sulphamethoxazole–trimethoprim and polymyxin B, while 87.5% were susceptible to tetracycline and ampicillin. These findings demonstrate the isolation of *E. coli* and *Salmonella* among diarrheic calves and highlight important calf‐management factors associated with bacterial isolation, as well as tetracycline resistance among *E. coli*. Improved calf management, appropriate colostrum feeding, adequate bedding, and prudent antimicrobial use are recommended. Further molecular characterization is warranted to determine the specific diarrheagenic *E. coli* pathotypes and clarify their contribution to calf diarrhea.

## 1. Introduction

Ethiopia possesses the largest livestock population in Africa, estimated at over 237 million head of livestock in recent years [1]. Dairy production is a key pillar of the livestock economy, providing essential food and income while offering strong growth opportunities due to increasing urban demand and favorable production conditions. However, despite this potential, intensive periurban dairy systems face challenges such as increased disease susceptibility, poor survival rates, and reduced reproductive performance [2].

Newborn calves are a vital component of livestock production, serving as future sources of meat and breeding stock and ensuring herd replacement and sustainability. Therefore, raising healthy calves is crucial for dairy and beef production systems. However, calf mortality can reach up to 50%, mainly due to poor management practices and the low adaptability of exotic breeds to tropical environments [3]. As a result, calfhood diseases have significant economic impacts through treatment costs, genetic losses, and reduced future productivity [4]. Among these diseases, diarrhea is one of the leading causes of morbidity and mortality in calves worldwide [5]. Its etiology is multifactorial, involving management, environmental, nutritional, and host‐related factors, as well as infectious agents such as bacteria, viruses, and parasites, which may act individually or in combination [6].

Bacterial infections play a major role in calf diarrhea, particularly in intensive production systems, leading to considerable economic losses due to mortality, treatment costs, and reduced growth performance [7, 8]. Among the pathogens, *Escherichia coli* and *Salmonella* species are the most important causes of bacterial diarrhea in calves [9, 10]. Diarrhea‐related morbidity and mortality have both immediate and long‐term impacts, including impaired growth and reduced herd replacement capacity [11]. These losses are further exacerbated by prolonged illness, poor thriftiness, and increased veterinary expenses [12]. Therefore, identifying the etiological agents involved in calf diarrhea is essential for designing effective control and prevention strategies [13, 14].

Current treatment of neonatal calf diarrhea primarily relies on antimicrobial and fluid therapy. However, the widespread and often indiscriminate use of antimicrobials has contributed to the emergence of antimicrobial resistance (AMR), which is a growing global concern. Resistance has been widely reported in *Salmonella* species and *E. coli*, particularly in preweaned calves [15]. In Ethiopia, improper drug use practices such as underdosing, overdosing, and lack of veterinary supervision further accelerate the development of multidrug‐resistant strains. Consequently, selecting effective antibiotics requires up‐to‐date knowledge of local resistance patterns.

Furthermore, incomplete treatment courses and the continuous misuse of antimicrobial drugs in both animals and humans pose serious public health risks, including drug residues and the spread of resistant bacteria. Although routine laboratory testing may be costly, periodic monitoring of antimicrobial susceptibility patterns is essential to guide effective treatment strategies [16]. In addition, identifying both etiological and predisposing factors is critical for improving livestock productivity and reducing early‐life losses [13, 17].

Dairy farming is a rapidly expanding sector in Ethiopia, particularly in urban and periurban areas where access to milk markets is better. The increasing use of exotic and crossbred dairy cattle has improved milk production but has also contributed to higher disease susceptibility. Calf morbidity and mortality due to diarrhea remain major constraints in these systems. Despite this, there is limited information on the prevalence, risk factors, and AMR patterns associated with calf diarrhea in Ethiopia. Therefore, this study aimed to isolate and identify *Salmonella* and *E. coli* from diarrheic calves, assess associated risk factors, and determine the antimicrobial susceptibility patterns of the isolates in and around Sebeta Town, Ethiopia.

## 2. Materials and Methods

### 2.1. Description of the Study Area

The study was conducted on diarrheic calves from November 2018 to March 2019 in and around Sebeta Town, a suburb of Addis Ababa located in the Oromia Special Zone surrounding Finfinne, Oromia Region, Ethiopia. Sebeta is situated at a latitude of 8°54′40″ N and longitude of 38°37′17″ E, at an elevation of 2356 m (7730 feet) above sea level. The area has an average annual temperature of 17.4°C and receives approximately 1650 mm of annual rainfall. The monthly precipitation averages about 150 mm during the wet season and falls below 30 mm during the dry season. The farming systems in the study area include intensive, semi‐intensive, and extensive systems; however, this study focused on the intensive farming system.

### 2.2. Study Population

The study population consisted of local and crossbred calves managed under intensive production systems in and around Sebeta Town. Farms and households were visited during the study period to assess the health status of the calves. During each visit, calf management practices, housing conditions, and sanitation status were observed. Sick animals were treated after clinical examination and after the necessary samples had been collected. Relevant data, including age, sex, body condition, breed, and manure management practices, were recorded during sample collection.

### 2.3. Study Design and Sampling Technique

A cross‐sectional study was conducted on small‐, medium‐, and large‐scale dairy farms in and around Sebeta Town from November 2018 to March 2019. The town and kebeles were selected purposively, while dairy farms were selected by the simple random sampling technique. Calves were chosen purposively based on clinical signs of diarrhea, including elevated body temperature, depression, dehydration, reduced suckling reflex, rough hair coat, weight loss, weakness, and soiling of the hindquarters and tail with diarrheic feces [18].

### 2.4. Fecal Sample Collection, Storage, and Transportation

A total of 129 fecal samples were collected purposively from calves less than two months of age showing clinical signs of diarrhea. Only calves that had not received antibiotic treatment for at least 2 weeks prior to sampling were included to minimize antimicrobial interference with bacterial isolation. Samples were collected directly from the rectum using sterile surgical gloves and placed into sterile, screw‐capped universal bottles. They were transported to the Microbiology Laboratory of the National Animal Health Institute (AHI) in an ice box maintained at approximately 4°C and processed within 3–4 h of collection. At the time of sampling, relevant animal data, including fecal consistency, age, breed, body condition, ear tag number, feeding practices, and history of diarrhea, were recorded using a standardized format. A calf was considered diarrheic if it passed semifluid to watery feces, with or without mucus and/or blood. Isolation and identification of *Salmonella* spp. and *E. coli* were performed using conventional bacteriological methods [19]. However, *Salmonella* isolates were not further characterized by serotyping due to laboratory limitations. Antimicrobial susceptibility testing was conducted using standard procedures, and multidrug resistance (MDR) was defined as resistance to three or more antimicrobial classes.

### 2.5. Isolation and Identification of *Salmonella*

Isolation of *Salmonella* was performed as follows: Approximately 1 g of feces was pre‐enriched in 9 mL of buffered peptone water (BPW) and incubated overnight at 37°C. From the pre‐enriched culture, 100 μL of suspension was transferred into 10 mL of Rappaport–Vassiliadis (RVS) enrichment broth (Oxoid, USA) and incubated at 42°C for 24 h. Cultures showing growth in RVS broth were streaked onto xylose lysine deoxycholate (XLD) agar (Oxoid, USA) and incubated at 37°C for 24–48 h. Colonies were examined for typical *Salmonella* morphology, characterized by red colonies with black centers due to hydrogen sulfide (H_2_S) production. Suspected colonies were subcultured on nutrient agar to obtain pure cultures and subjected to biochemical tests, including the triple sugar iron (TSI) test, citrate utilization test, urease test, indole test, methyl red test, and Voges–Proskauer test. Final confirmation and subspecies identification were performed using a Biolog (GENE III) semiautomated system.

### 2.6. Isolation and Identification of *E. coli*

For the isolation of *E. coli*, fecal samples were inoculated onto MacConkey agar and eosin methylene blue (EMB) agar and incubated at 37°C overnight. Growth on MacConkey agar was used as a preliminary criterion for identification. Colonies were classified based on lactose fermentation: Lactose‐fermenting colonies (pink) were considered presumptive *E. coli*. These colonies were further subcultured onto EMB agar for confirmation. Colonies showing a characteristic metallic green sheen or blue–black coloration on EMB agar were considered indicative of *E. coli*. Further confirmation was performed using biochemical tests, including TSI, citrate utilization, urease, indole, methyl red, and Voges–Proskauer tests [20–22]. Pure colonies were obtained by subculturing on nutrient agar. Confirmed *E. coli* isolates were preserved on brain heart infusion (BHI) agar slants and stored until antimicrobial susceptibility testing. Confirmation of isolates was also performed using a Biolog semiautomated system.

However, it is important to note that the isolation and identification procedures employed in this study do not differentiate between pathogenic and commensal *E. coli* strains. As *E. coli* is a normal inhabitant of the gastrointestinal tract, its recovery from fecal samples does not necessarily indicate a causal role in diarrhea. The study did not include molecular or serological characterization of virulence factors such as F5 (K99), F17, STa, STb, LT, or intimin, which are required to confirm enterotoxigenic (*ETEC*) or attaching/effacing (*AEEC*) pathotypes. Therefore, the reported isolation rate reflects the presence of *E. coli* in diarrheic calves rather than definitive attribution as the etiological agent. This limitation should be considered when interpreting the findings, and future studies incorporating virulence gene detection are recommended to establish pathogenicity.

### 2.7. Antimicrobial Susceptibility Test

Antimicrobial susceptibility testing was performed using the standard Kirby–Bauer agar disk diffusion method according to the Clinical and Laboratory Standards Institute (CLSI) veterinary guidelines [23]. The selection of antibiotics was based on their common use in ruminants, public health importance, and standard antimicrobial susceptibility testing guidelines. Pure bacterial colonies were inoculated into tryptone soya broth (Oxoid, England) and incubated for 6 h. The bacterial suspension was adjusted to match the turbidity of a 0.5 McFarland standard and inoculated onto Mueller–Hinton agar plates (Becton Dickinson, USA). Antibiotic disks were placed on the inoculated plates, which were then incubated at 37°C for 18–24 h. Zone diameters were measured and interpreted as susceptible, intermediate, or resistant according to the CLSI Veterinary Performance Standards for Antimicrobial Disk and Dilution Susceptibility Tests for Bacteria Isolated from Animals (VET01S) [23]. The following antibiotic disks were used: gentamicin (10 μg), ampicillin (10 μg), chloramphenicol (30 μg), sulfamethoxazole–trimethoprim (25 μg), polymyxin B, and tetracycline (30 μg).

In this study, chloramphenicol was included solely as a reference antimicrobial for comparative analysis and epidemiological surveillance of AMR. Therefore, the susceptibility results for chloramphenicol should be interpreted only in the context of resistance monitoring and should not be considered a recommendation for its clinical use in food‐producing animals, where its use is prohibited or strictly restricted in many countries. Future studies should prioritize antimicrobials that are approved for veterinary use and have direct clinical relevance in food‐producing animals.

### 2.8. Data Management and Analysis

Data generated from the study were coded, organized, and entered into Microsoft Excel (Microsoft Office Excel 2007). The prevalence of *Salmonella* and *E. coli* was calculated as the proportion of culture‐positive samples out of the total number of samples examined. Statistical analysis was performed using STATA Version 12. Descriptive statistics were used to summarize the characteristics of the study variables. Categorical variables were presented using frequencies and percentages. The association between each independent variable and *E. coli* infection was initially assessed using univariable logistic regression analysis. Variables with a *p*‐value < 0.25 in the univariable analysis were subjected to inclusion in the multivariable logistic regression model. This relatively liberal significance level was used to avoid excluding potentially important variables that could become significant after adjustment for other factors.

Before fitting the multivariable model, the selected independent variables were assessed for multicollinearity. The correlation between independent variables was examined, and variables showing a strong correlation (*r* ≥ +0.60 or *r* ≤ −0.60) were considered highly correlated. Where substantial correlation was identified, variables were carefully evaluated, and the most epidemiologically relevant variable was retained in the multivariable model to avoid redundancy and instability in the estimates.

Multivariable logistic regression analysis was then conducted to identify factors independently associated with *E. coli* infection while controlling for the effects of other variables in the model. A variable was considered a statistically significant predictor of *E. coli* infection when the *p*‐value was < 0.05 and the corresponding 95% CI for the OR did not include one.

Potential confounding was assessed by comparing the odds ratios obtained before and after adjustment. A variable was considered to be an important confounder if its inclusion in the model resulted in a 20% or greater change in the odds ratio of the principal exposure variable. Potential confounding variables were therefore retained in the final model when they met this criterion, even if they were not statistically significant. Model adequacy and goodness‐of‐fit were assessed using appropriate model diagnostic procedures, including the Hosmer–Lemeshow goodness‐of‐fit test. The model was considered to have an adequate fit when the Hosmer–Lemeshow test was not statistically significant (*p* > 0.05). The final results were presented as adjusted odds ratios (AORs) with their corresponding 95% confidence intervals and *p*‐values. Statistical significance was set at a *p*‐value of < 0.05, with a 95% confidence level.

## 3. Results

### 3.1. Overall Prevalence and Distribution of Bacterial Isolates

Out of the 129 fecal samples examined, 65 (50.3%) (95% CI: 41.6–59.1) were positive for *E*. *coli*, while 8 (6.2%) (95% CI: 3.1–12.01) were positive for *Salmonella*, indicating that *E. coli* was the predominant bacterial isolate from calf diarrhea in the study area (Table 1).

**TABLE 1:** The overall isolation of *E. coli* and *Salmonella* isolated from diarrheic calves in and around Sebeta Town.

Isolates	Sample type	Total number examined	Number of +ve (prevalence)	95% confidence interval
*E. coli*	Feces	129	65 (50.3%)	41.6–59.1
*Salmonella*	Feces	129	8 (6.2%)	1.98–10.4

Sex, age, bedding, and calf housing were significantly associated (*p* *< *0.05) with *Salmonella* isolation from diarrheic calves in the multivariable analysis. Those variables with *p* < 0.25 in univariable logistic regression analysis were subjected to multivariable logistic regression, and using the backward elimination technique, the final model was developed. Male calves had significantly higher odds of *Salmonella* isolation than female calves (OR = 9.85). Calves aged 6–8 weeks also had significantly higher odds of *Salmonella* isolation compared with those older than 2 weeks (OR = 77.02). Similarly, calves kept without bedding had significantly higher odds of *Salmonella* isolation than those provided with bedding (OR = 12.09). In contrast, calves housed in pens had significantly lower odds of *Salmonella* isolation compared with those housed in the same barn (OR = 0.09) (Table 2).

**TABLE 2:** Association between risk factors and isolated *Salmonella* from diarrheic calves (*n* = 129).

Risk factor	Category	Total sample	No. of positive	Univariable		Multivariable	
				OR (95% CI)	*p*‐value	OR (95% CI)	*p*‐value
Farm size	Small	17	1	Ref	—	—	—
	Medium	33	3	1.60 (0.15–16.66)	0.694	—	—
	Large	79	4	0.85 (0.09–8.15)	0.890	—	—

Sex	Female	78	3	Ref	—	Ref	—
	Male	51	5	2.72 (0.62–11.91)	0.185	9.85 (1.42–68.31)	0.021

Age (wk)	> 2 weeks	69	3	Ref	—	Ref	—
	2–4 weeks	47	4	2.05 (0.44–9.60)	0.364	2.85 (0.47–17.07)	0.252
	4–6 weeks	11	0	—	—	—	—
	6–8 weeks	2	1	22.00 (1.09–443.48)	0.044	77.02 (1.40–4229.42)	0.034

Colostrum feeding	> 6 h	86	2	Ref	—	—	—
	6–12 h	28	4	7.00 (1.21–40.56)	0.030	—	—
	> 24 h	15	2	6.46 (0.84–49.95)	0.074	—	—

Bedding	Bedding	60	1	Ref	—	Ref	—
	No bedding	69	7	6.66 (0.80–55.80)	0.080	12.09 (1.11–131.47)	0.041

House hygiene	Poor	49	3	Ref	—	—	—
	Good	80	5	1.02 (0.23–4.48)	0.977	—	—

Breed	Cross	3	0	—	—	—	—
	Local	126	8	—	—	—	—

Calf housing	Same barn	63	1	Ref	—	Ref	—
	Pen	66	7	0.14 (0.02–1.14)	0.066	0.09 (0.01–0.87)	0.038

Variables with *p* < 0.25 in the univariable logistic regression analysis were considered for inclusion in the multivariable model, and backward elimination was used to develop the final model. In the multivariable analysis, significant associations with *E. coli* isolation were observed for sex, age, colostrum‐feeding time, bedding provision, and calf housing (*p* < 0.05). Male calves had significantly higher odds of *E. coli* isolation than female calves (adjusted OR = 7.42). Calves aged 4–6 weeks had significantly higher odds of *E. coli* isolation compared with calves aged < 2 weeks (adjusted OR = 15.10), whereas calves aged 2–4 weeks did not differ significantly from the reference group. Calves receiving colostrum at 6–12 h after birth had significantly higher odds of *E. coli* isolation than those receiving colostrum within < 6 h (adjusted OR = 3.47), while the association for calves receiving colostrum after > 24 h was not statistically significant. Calves kept without bedding had significantly higher odds of *E. coli* isolation than those provided with bedding (adjusted OR = 4.74). Similarly, calves housed in pens had significantly higher odds of *E. coli* isolation than those housed in the same barn (adjusted OR = 2.42) (Table 3).

**TABLE 3:** Association between risk factors and isolated *E. coli* from diarrheic calves (*n* = 129).

Risk factor	Category	Total sample	No. of positive	Univariable OR (95% CI)	*p*‐value	Multivariable OR (95% CI)	*p*‐value
Farm size	Small	17	9	Ref	—	—	—
	Medium	33	20	1.37 (0.42–4.45)	0.603	—	—
	Large	79	36	0.74 (0.26–2.13)	0.581	—	—

Sex	Female	78	30	Ref	—	Ref	—
	Male	51	35	3.50 (1.66–7.39)	0.001	7.42 (2.83–19.44)	< 0.001

Age (wk)	< 2 weeks	69	28	Ref	—	Ref	—
	2–4 weeks	47	26	1.81 (0.86–3.84)	0.120	1.73 (0.71–4.20)	0.228
	4–6 weeks	11	9	6.59 (1.32–32.83)	0.021	15.10 (2.13–106.77)	0.007
	6–8 weeks	2	2	—	—	—	—

Colostrum feeding	< 6 h	86	36	Ref	—	Ref	—
	6–12 h	28	19	2.93 (1.19–7.22)	0.019	3.47 (1.16–10.37)	0.026
	> 24 h	15	10	2.78 (0.87–8.82)	0.083	3.09 (0.65–14.77)	0.158

Bedding	Bedding	60	23	Ref	—	Ref	—
	No bedding	69	42	2.50 (1.23–5.09)	0.011	4.74 (1.86–12.09)	0.001

House hygiene	Poor	49	29	Ref	—	Ref	—
	Good	80	36	0.56 (0.27–1.16)	0.119	—	—

Breed	Cross	3	2	Ref	—	—	—
	Local	126	63	0.50 (0.04–5.66)	0.575	—	—

Calf housing	Same barn	63	27	Ref	—	Ref	—
	Pen	66	38	0.55 (0.27–1.11)	0.096	2.42 (1.01–5.78)	0.047

### 3.2. Antimicrobial Susceptibility Patterns of *Salmonella* and *E. coli*

The antimicrobial susceptibility testing revealed that all *Salmonella* isolates (100%) were highly susceptible to sulfamethoxazole–trimethoprim and polymyxin B. High levels of susceptibility (87.5%) were also observed for ampicillin, chloramphenicol, and tetracycline, while gentamicin showed comparatively lower susceptibility (75%). Only one isolate (12.5%) exhibited resistance to tetracycline, indicating generally low levels of AMR among *Salmonella* isolates. In contrast, MDR was observed among *E. coli* isolates. The most common resistance patterns involved tetracycline in combination with other antibiotics, particularly tetracycline + polymyxin B (25%) and tetracycline + gentamicin (20%), with other combinations involving ampicillin, chloramphenicol (used as reference/surveillance for AMR test), and sulfamethoxazole–trimethoprim occurring at lower frequencies. Overall, resistance to tetracycline was the most frequently observed pattern. Regarding the antimicrobial susceptibility profile of *E. coli*, high susceptibility was recorded for sulfamethoxazole–trimethoprim (93.85%), chloramphenicol (87.7%), polymyxin B (78.5%), gentamicin (76.92%), and ampicillin (73.85%). However, a high level of resistance was observed for tetracycline (58.46%), followed by polymyxin B (21.54%), ampicillin (12.31%), and gentamicin (9.23%). Among the tested antibiotics, tetracycline exhibited the highest level of resistance (Table 4).

**TABLE 4:** Antimicrobial susceptibility pattern of *Salmonella* isolates to tested drugs.

Antimicrobial agents	No. of susceptible (%)	No. of intermediate (%)	No. of Resistant (%)
Tetracycline	7 (87.5%)		1 (12.5%)
Gentamicin	6 (75%)	1 (12.5%)	1 (12.5%)
Chloramphenicol	7 (87.5%)		1 (12.5%)
Polymixin B	8 (100%)		0
Ampicillin	7 (87.5)	1 (12.5%)	
Sulphamethoxazole–trimethoprim	8 (100%)		

The MDR analysis revealed that *E. coli* isolates from diarrheic calves exhibited varying resistance patterns involving two to three antimicrobial agents. Out of the 65 *E. coli* isolates recovered, 20 isolates exhibited resistance to two or more antimicrobial agents and were therefore included in the multidrug resistance‐pattern analysis (Table 5). The most frequent resistance patterns involved tetracycline, particularly tetracycline–polymyxin B (25.0%) and tetracycline–gentamicin (20.0%), indicating that tetracycline‐based resistance was predominant. Although both two and three‐drug resistance patterns were observed, according to the predefined MDR criterion of resistance to at least one antimicrobial agent in three or more antimicrobial classes, only three of the twenty multiresistant isolates (15.0%) fulfilled the MDR definition. These MDR patterns included tetracycline–ampicillin–polymyxin B, tetracycline–polymyxin B–gentamicin, and tetracycline–chloramphenicol–ampicillin. The remaining multiresistant isolates exhibited resistance to only two antimicrobial agents and were therefore not classified as MDR according to the predefined criterion. The antimicrobial susceptibility profile showed varying levels of effectiveness among the tested antimicrobial agents. High susceptibility was observed for sulphamethoxazole–trimethoprim (93.85%), chloramphenicol (used as reference/surveillance for AMR test 87.70%), and polymyxin B (78.46%). Gentamicin and ampicillin also showed moderate effectiveness, whereas tetracycline exhibited the highest resistance rate (Table 6).

**TABLE 5:** Multidrug resistance patterns among *E. coli* isolates exhibiting resistance to two or more antimicrobial agents among diarrheic calves (*n* = 20).

Frequency	Drugs	Percentages (%)
1	Tetra, sulpha–trime	5%
1	Tetra, Ampi	5%
1	Tetra, Ampi, PB	5%
1	Tetra, PB, Gent	5%
1	Tetra, chlorop, Ampi	5%
2	ST, Amp	10%
2	Ampi, PB	10%
2	Tetra, chlorop	10%
4	Tetra, Gent	20%
5	Tetra, PB	25%
20		100%

**TABLE 6:** Antimicrobial susceptibility pattern of *E. coli* isolated from diarrheic calves (*n* = 65).

Antimicrobial agent	Susceptible *N* (%)	Intermediate *N* (%)	Resistant *N* (%)
Tetracycline	27 (41.54%)	0 (0.00%)	38 (58.46%)
Gentamicin	50 (76.92%)	9 (13.85%)	6 (9.23%)
Chloramphenicol	57 (87.70%)	4 (6.15%)	4 (6.15%)
Polymyxin B	51 (78.46%)	0 (0.00%)	14 (21.54%)
Ampicillin	48 (73.85%)	9 (13.85%)	8 (12.31%)
Sulphamethoxazole–trimethoprim	61 (93.85%)	0 (0.00%)	4 (6.15%)

## 4. Discussion

The present study was conducted to isolate and identify *Salmonella* and *E*. *coli* from diarrheic calves, assess associated risk factors, and determine the antimicrobial susceptibility patterns of the isolates. The isolation rate of *E. coli* (50.3%) observed in this study was consistent with the findings of Reference [24] (44% [25], 43.1%); however, the present result was higher than the reports of Reference [9, 26–28], which documented isolation rates of 23%, 25%, 49%, and 38.6%, respectively, in different countries. However, it was lower than the findings of Reference [29–33], which reported higher isolation rates of 75%, 76%, 70.7%, 76%, and 60% from various locations. The variation among studies may be attributed to differences in study design, geographical location, farm management practices, calf age, sampling methods, and diagnostic approaches. However, since *E. coli* is a normal intestinal commensal, its isolation from diarrheic fecal samples alone does not confirm its role as a diarrheal pathogen. The finding that *E. coli* was predominately recovered from diarrheic calves in the present study may be an indication of its importance in calf diarrhea in the study area. Virulence‐associated factors and diarrheagenic *E. coli* pathotypes were not characterized; therefore, the isolated *E. coli* was interpreted as being associated with diarrheic calves rather than confirmed as the causative agent of diarrhea. Furthermore, neonatal calf diarrhea is a multifactorial condition involving a complex interaction of bacterial, viral, protozoal, nutritional, and management‐related factors. Although this study investigated two important bacterial pathogens, *E. coli* and *Salmonella*, other significant enteric pathogens were not assessed. Therefore, the potential contribution of these pathogens, as well as mixed infections, to the occurrence of calf diarrhea could not be excluded.

The prevalence of *Salmonella* (6.2%) observed in this study was lower than the reports of Reference [32] (13%) and [9] (21.9%) but consistent with the findings of References [34] (9.61%), [35] (4%), and [26] (5%). These variations in prevalence between studies may be attributed to differences in sample size, calf management practices, environmental conditions, and distribution of risk factors such as age, sex, and farm size. In the present study, although a higher proportion of diarrhea cases were observed in large‐scale farms (61.24%) compared to medium (27.13%) and small farms (13.18%), the difference was not statistically significant. This may suggest that factors other than farm size, such as management practices and hygiene, play a more critical role.

Age was significantly associated with the isolation of both *E. coli* and *Salmonella*. In the present study, the highest odds of *E. coli* isolation were observed among calves aged 4–6 weeks, while calves aged 6–8 weeks showed higher odds of *Salmonella* isolation compared with the reference age group. This finding was aligned with the reports of References [36, 37], which stated that younger calves, particularly those below 1 month of age, have been reported to have a higher occurrence of *E. coli*, reflecting their greater susceptibility to enteric infections. In contrast, *Salmonella* occurrence has been reported to be higher among somewhat older calves, particularly those approximately 2–4 months of age [38]. In the present study, however, the variation in *Salmonella* occurrence among age categories was not considered significant or indicative of a general age‐related pattern. This variation may be attributed largely to the unequal number of calves sampled across the different age groups, particularly the small number of calves in the 6–8‐week age category. Age was significantly associated with the isolation of both *E. coli* and *Salmonella*.

The timing of colostrum feeding was also identified as a significant risk factor for both *E. coli* isolation and *Salmonella*. Calves that received colostrum later than 6 hours after birth were more likely to develop diarrhea. This finding was consistent with previous reports indicating that each hour of delay in colostrum ingestion within the first 12 h of life increases the likelihood of illness by approximately 10%. It was reported that about 61% of colostral immunoglobulins (containing approximately 80 mg/mL of IgG) were absorbed within the first 6 hours after birth, after which absorption declines sharply [39]. Therefore, delayed colostrum intake may lead to inadequate passive transfer of maternal immunity, increasing susceptibility to enteric infections [40, 41].

Sex was also significantly associated with the isolation of *E. coli*, with male calves showing higher prevalence than females. This difference may be attributed to management practices, where female calves are often given more attention due to their future role in herd replacement, while male calves may receive relatively less care.

Housing conditions and management practices were also found to influence the occurrence of bacterial isolation. Although the association was not statistically significant, a higher proportion was observed in calves reared under intensive management systems compared to those in extensive systems. This could be due to increased animal density, poor hygiene, and greater environmental contamination, which facilitate the transmission of enteric microorganisms. Similar observations have been reported, where indoor housing and the use of processed feed may increase exposure to bacterial organisms [11]. Additionally, livestock waste from animals fed predominantly on grass has been reported to harbor fewer *Salmonella* organisms [42]. The higher occurrence of bacterial isolates in calves with severe diarrhea may suggest a possible association between these organisms and intestinal disturbance; however, causation cannot be established in this study, as other important enteric pathogens such as rotavirus, coronavirus, and *Cryptosporidium* spp. were not investigated.

The antimicrobial susceptibility results showed that *E. coli* isolates were highly susceptible to chloramphenicol and sulfamethoxazole–trimethoprim, which was consistent with findings reported by References [31, 33]. However, it should be noted that chloramphenicol use in food‐producing animals is banned or highly restricted in many countries due to public health concerns; therefore, these findings were interpreted, not directly translated, into treatment recommendations.

A high level of resistance to tetracycline (58.46%) was observed in the present study, which contrasts with findings by References [27, 28], which reported resistance levels exceeding 80%, and Hossain et al. (2012), which documented 100% sensitivity to tetracycline. These discrepancies may be due to differences in antimicrobial usage practices and the emergence of resistant strains in different geographical regions. Furthermore, the presence of multidrug‐resistant *E. coli* isolates in this study indicated a growing concern regarding AMR, likely driven by the indiscriminate use of antibiotics in livestock production. In this study, MDR was defined as resistance to three or more antimicrobial classes**.** In contrast, *Salmonella* isolates showed relatively low resistance, with most isolates being susceptible to the tested antimicrobials.

In conclusion, the study demonstrated that *E. coli* (50.3%) and *Salmonella* (6.2%) were isolated from fecal samples of diarrheic calves in and around Sebeta Town, with *E. coli* being the predominant isolate. However, the isolation of *E. coli* from diarrheic calves should not be interpreted as definitive evidence that the organism caused the diarrhea, since virulence characteristics and other potential etiological agents were not investigated. Several calf‐level and management factors, including sex, age, timing of colostrum feeding, bedding provision, and housing system, were significantly associated with the isolation of these bacteria. Antimicrobial susceptibility results showed that most isolates were sensitive to sulfamethoxazole–trimethoprim and chloramphenicol; however, resistance to commonly used drugs, especially tetracycline, and the presence of MDR among *E. coli* isolates were observed. Therefore, improving colostrum management, bedding, housing, hygiene, and overall calf management may help reduce the risk of enteric bacterial infection and calf diarrhea. Rational use of antimicrobials based on susceptibility testing and increased farmer awareness is strongly recommended to reduce calf diarrhea and AMR. Further studies incorporating molecular characterization of virulence factors and investigation of other infectious and non‐infectious causes of calf diarrhea are recommended to establish the specific contribution of *E. coli* and *Salmonella* to disease in the study area.

### 4.1. Limitations of the Study

This study has some limitations that should be considered when interpreting the findings. Although *E*. *coli* was isolated from diarrheic calves, no virulence characterization was performed; therefore, it is not possible to determine whether the isolates were pathogenic or commensal strains. Only eight *Salmonella* isolates were recovered. This relatively small number limits the statistical power for evaluating associated risk factors and interpreting antimicrobial susceptibility patterns. Consequently, the findings related to *Salmonella* should be interpreted with caution. The study was based on samples collected between 2018 and 2019. Changes in management practices, antimicrobial usage, and pathogen dynamics over time may affect the current relevance of the findings.

## Author Contributions

Conceptualization: Atinafu Regasa Hailu, Fikru Gizaw Gurmessa, and Shubisa Abera Leliso; data curation: Atinafu Regasa Hailu, Fikru Gizaw Gurmessa, and Shubisa Abera Leliso; formal analysis: Teshita Edaso Bariso, Fikru Gizaw Gurmessa, and Shubisa Abera Leliso; investigation: Atinafu Regasa Hailu and Shubisa Abera Leliso; methodology: Atinafu Regasa Hailu, Fikru Gizaw Gurmessa, Teshita Edaso Bariso, and Shubisa Abera Leliso; writing–original draft: Atinafu Regasa Hailu and Teshita Edaso Bariso; writing–review and editing: Teshita Edaso Bariso and Fikru Gizaw Gurmessa.

## Funding

The authors received no specific funding for this work.

## Ethics Statement

Permission for ethical approval was obtained from the College of Veterinary Medicine, Samara University. Fecal samples were collected following prior explanation of the objectives of the study to the dairy farm owners and sampled based on their consent.

## Conflicts of Interest

The authors declare no conflicts of interest.

## Data Availability

The datasets used and/or analyzed during the current study are available from the corresponding author on reasonable request.
